# *Ld*Flabarin, a New BAR Domain Membrane Protein of *Leishmania* Flagellum

**DOI:** 10.1371/journal.pone.0076380

**Published:** 2013-09-27

**Authors:** Michèle Lefebvre, Emmanuel Tetaud, Magali Thonnus, Bénédicte Salin, Fanny Boissier, Corinne Blancard, Cécile Sauvanet, Christelle Metzler, Benoît Espiau, Annelise Sahin, Gilles Merlin

**Affiliations:** 1 CNRS UMR 5290, Montpellier, France; 2 Université Montpellier 1, Montpellier, France; 3 Centre Hospitalier Universitaire La Colombière, Montpellier, France; 4 IRD 224, Montpellier, France; 5 CNRS UMR 5095, Institut de Biochimie Génétique et Cellulaire, Bordeaux, France; 6 CNRS UMR 5234, Bordeaux, France; 7 Université Bordeaux Segalen, Bordeaux, France; 8 CNRS-EPHE USR 3278, Papetoai, Moorea, Polynésie Française; Universidad Nacional Autónoma de México, Mexico

## Abstract

During the *Leishmania* life cycle, the flagellum undergoes successive assembly and disassembly of hundreds of proteins. Understanding these processes necessitates the study of individual components. Here, we investigated *Ld*Flabarin, an uncharacterized *L. donovani* flagellar protein. The gene is conserved within the *Leishmania* genus and orthologous genes only exist in the *Trypanosoma* genus. *Ld*Flabarin associates with the flagellar plasma membrane, extending from the base to the tip of the flagellum as a helicoidal structure. Site-directed mutagenesis, deletions and chimera constructs showed that *Ld*Flabarin flagellar addressing necessitates three determinants: an N-terminal potential acylation site and a central BAR domain for membrane targeting and the C-terminal domain for flagellar specificity. In vitro, the protein spontaneously associates with liposomes, triggering tubule formation, which suggests a structural/morphogenetic function. *Ld*Flabarin is the first characterized *Leishmania* BAR domain protein, and the first flagellum-specific BAR domain protein.

## Introduction

Eukaryotic flagella present a remarkable evolutionary conservation of their structure and constituents [1]. They have been mostly studied in *Chlamydomonas reinhardtii* [2], [3] and *Trypanosoma brucei* [4], [5], two flagellated organisms. Thus, intraflagellar transport was first described in *C. reinhardtii* before being generalized to all eukaryotic flagella and cilia [6], [7]. Stressing their practical importance, protozoan studies allowed the identification of human orthologue genes whose mutations are responsible for pleiotropic, severe genetic diseases, such as polycystic kidney disease or Bardet-Biedl syndrome [8].

*Leishmania* are flagellated protozoan kinetoplastid parasites [9]. They exist alternatively as amastigotes (intracellular mammalian forms) and promastigotes (extracellular insect forms) [10]. While the amastigote flagellum barely sticks out of the cell body, its promastigote counterpart may be twice as long as the cell body. The flagellum is essential for promastigote motility within the insect digestive tract, allowing the migration of the parasites to the mouth parts and hence their transmission to a mammalian host through biting [11]. Recent elegant experiments have also shown that the flagellum is indispensable for infectious promastigotes to infect mammalian host macrophages where they transform into amastigotes [12]. Moreover, the flagellum is also suspected to bear other essential functions like cellular organization and sensory perception [13]. The differentiation from amastigotes to promastigotes and reciprocally requires the successive assembly and disassembly of hundreds of proteins. The dynamics of these processes is tightly regulated and their understanding necessitates the characterization of individual elements.

Bin/Amphiphysin/Rvs (BAR) domains are 200-amino-acid modular elements found in many eukaryotic multi-domain proteins [14]. Although their amino acid sequence may be poorly conserved and therefore difficult to identify, their structure is well conserved throughout evolution [15]. The archetype BAR domain consists of a monomer of three α-helices folded onto each other. Dimers form banana-shaped molecules with a positively charged concave face that interacts with the negatively charged lipid membranes [16]. BAR domains recognize or generate membrane curvature by inserting into the lipid bilayer. The specific membrane to which they bind to depends on adjacent domains, e.g., PH domains [17]. Since their discovery [18], BAR domains revealed diverse and have been categorized into several families and sub-families; some associate with concave, others with convex membranes [19]. BAR domain proteins are involved in membrane shaping, in the formation of endocytosis vesicles, tubules, endosomes, T-tubules, podosomes, filopodia, mitochondria and autophagosomes [20].

Here we report the characterization of *Ld*Flabarin (*L. donovani* FLAgellar BAR domain proteIN). To our knowledge, it is the first BAR domain protein associated with a eukaryotic flagellum and the first BAR domain protein found in *Leishmania*. We show that its flagellar addressing depends on several determinants and presents some originality within the genus *Leishmania* and the BAR domain superfamily. It arranges into a helicoidal structure around the flagellum and provokes the tubulation of artificial membranes in vitro. This suggests a -role in flagellar morphogenesis or structural stability.

## Results

### Identification of the *Leishmania* Flabarin

We previously discovered the involvement of small G proteins in the biogenesis of the *Leishmania* flagellum [21]–[23], which led us to try and identify effectors. One of the approaches used was bioinformatics: the *L. major* genome [24], the only available *Leishmania* genome sequence at the time of the experiments, was searched for homologues of known partners of the human ARF/ARL small G proteins [25]–[27].

We found *Lmj*F.27.1730, an unannotated 340-aa protein, as potential homologue of human *Hs*Arfaptin-1 (Genbank U52521), a partner of several ARF/ARL family members [25], [28]. The N-terminal region of *Lmj*F.27.1730 (amino acids 10–231) showed some identity (Fig. S1A) with the C-terminal region of *Hs*Arfaptin-1 (amino acids 61–339 for a total length of 341). The presence of a polypyrimidine tract 59−12 nt upstream of the start codon, followed by an AG dinucleotide (7−6 nt upstream of the start codon), a potential spliced leader attachment site [29], validated the predicted functionality of the *Lmj*F.27.1730 ORF.

For consistency with our previous work, we chose to focus on *L. donovani*. The orthologue was PCR amplified from *L. donovani* LV9 genomic DNA, using oligonucleotides designed from the *Lmj*F.27.1730 sequence. The *L. donovani* protein comprised 339 amino acids, with a predicted molecular mass of 37 827 Da and a pI of 5.17. Its amino acid sequence was 90% identical to *Lmj*F.27.1730 and differed by one amino acid with the now available *L. infantum* orthologue sequence (*Lin*J.27.1630) (Fig. S1B). For reasons becoming obvious below, these new proteins were named Flabarins for FLAgellar BAR domain proteINs.

### Phylogenetic analysis of Flabarins

Blast searches and available data [24] revealed the existence of *Ld*Flabarin orthologues in trypanosomatids (Fig. S1B). Flabarins amino acid sequences and lengths were well conserved within the genus *Leishmania* (*L. infantum*, *L. major*, *L. mexicana*, and *L. braziliensis*): 339–340 amino acids, 99.7−73.5% identity with *Ld*Flabarin. By contrast, *Trypanosoma* (*T. cruzi*, *T. vivax*, *T. congolense*, and *T. brucei*) Flabarins were shorter (222–269 amino acids) and more divergent (18–20% identity). Synteny was observed within these species [24] except for *T. vivax* (though *Tv*Flabarin sequence did not differ much from the other *Trypanosoma* Flabarin sequences). The *T. brucei* and *T. gambiense* Flabarin sequences were identical and the most divergent (18% identity). To our knowledge, the only previous report of trypanosomatid Flabarin is that of *T. brucei*, identified as a flagellar protein (*Tb*927.11.2410, formerly *Tb*11.22.0001) in the flagellome [4], [5] and not further studied. There is no apparent homologue in any other sequenced genome (Group OG5_148786 [30]), which makes the study of Flabarins particularly interesting.

### *Ld*Flabarin is a flagellar protein

**Figure 1:**
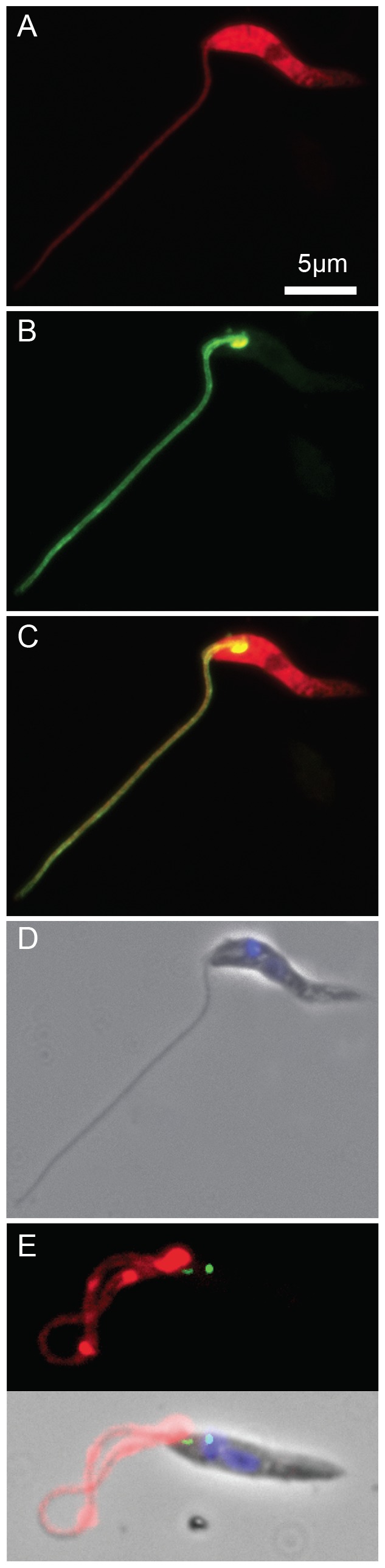
Intracellular localization of *Ld*Flabarin. *L. amazonensis* BA125 cells were co-transfected with pNUS mRednD-*Ld*Flabarin and pNUS *Ld*Flabarin-GFPcH. (A–B) Fluorescence images from a cell expressing both mRed-*Ld*Flabarin (red)(A) and *Ld*Flabarin-GFP (green) (B). (C) Overlay of A and B. (D) Overlay of DAPI staining (blue) and phase contrast. (E) A cell expressing *Ld*Centrin-GFP (green) and *Ld*Flabarin-mRed.

*Ld*Flabarin intracellular localization was investigated in *L. amazonensis* BA125 cells co-expressing mRed-*Ld*Flabarin (with a free *Ld*Flabarin C-terminus) and *Ld*Flabarin-GFP (with a free *Ld*Flabarin N-terminus). Fluorescence microscopy showed that mRed-*Ld*Flabarin was diffusely distributed throughout the cell (Fig. 1A,C,D), while *Ld*Flabarin-GFP localized exclusively to the flagellum (Fig. 1B,C,D). About 2% of *Ld*Flabarin-GFP-expressing cells displayed a stronger fluorescence signal at the base of the flagellum (Fig. 1B), an area different from the basal body, as revealed by the marker *Ld*Centrin-GFP [31] (Fig. 1E) and much larger.

**Figure 2:**
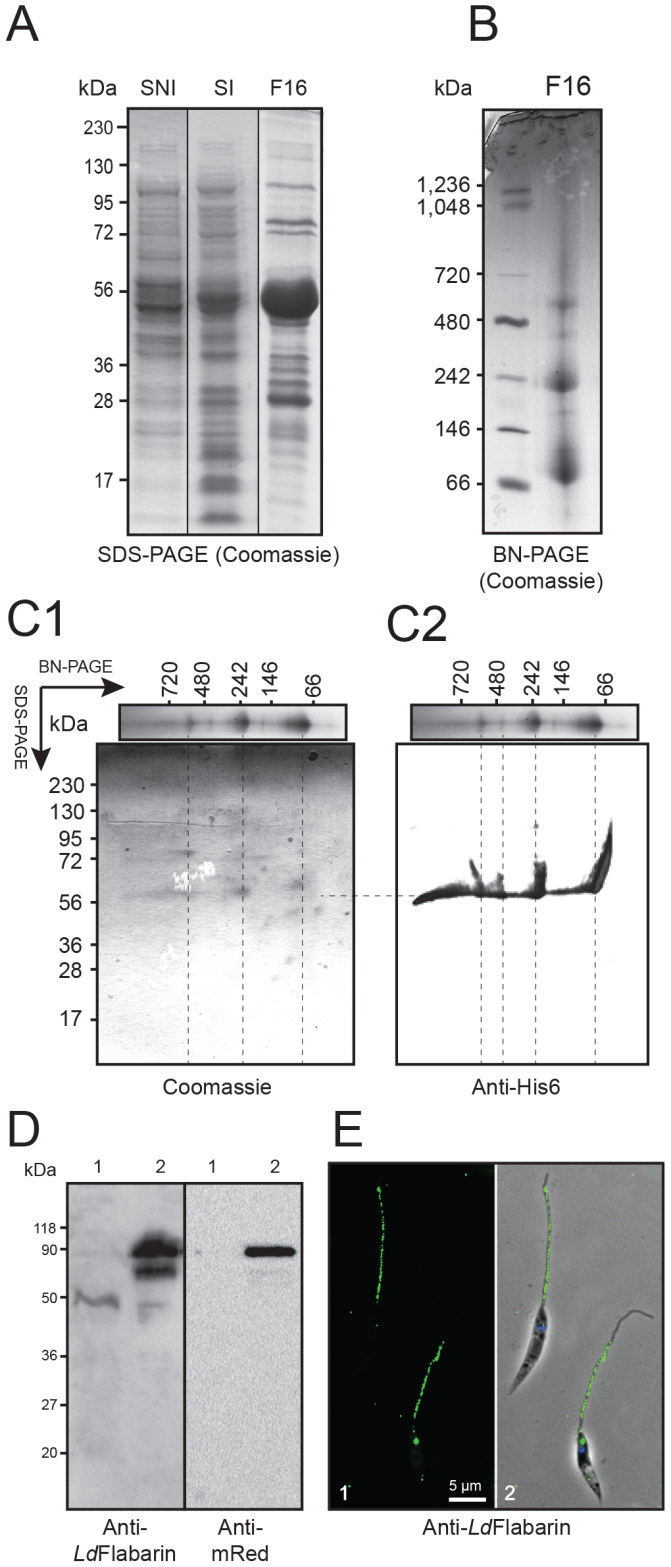
Production of recombinant *Ld*Flabarin-His6 and localization of *La*Flabarin in *Leishmania*. (A) 5 µg supernatant of non-induced (SNI), IPTG-induced (SI) and purified *Ld*Flabarin-His6 (F16) were separated by SDS-PAGE (denaturating polyacrylamide gel electrophoresis). (B) The purified fraction (F16) was submitted to a blue-native gel (BN-PAGE) allowing separation of complex. (C) The complex were separated by electrophoresis in first non-denaturing conditions (BN-PAGE), and then the track was subjected to a second electrophoresis under denaturing conditions (SDS-PAGE) to separate the components of the differents complex. The gel was stained with Coomassie (C1) or transferred to membranes and revealed with 1∶10000 anti-His (C2). (D) 3.10^6^ *L. amazonensis* BA125 untransfected control cells (lanes 1) and *Ld*Flabarin-mRed-expressing cells (lanes 2) were submitted to SDS-PAGE, transferred to membranes and revealed with 1∶2000 anti-*Ld*Flabarin (left panel) or 1∶10000 anti-mRed (right panel) and 1∶2500 anti-rabbit IgG conjugate as in [59]. (E) Localization of native *La*Flabarin in *L. amazonensis* BA125 by indirect immunofluorescence. Cells were fixed and incubated with anti-*Ld*Flabarin (1∶1000) and Alexa-labelled anti-rabbit IgG (8 µg/ml). Panel 1, *La*Flabarin green fluorescence image of two cells in the same field; panel 2, overlay of panel 1 with DAPI staining (blue) and phase contrast.

Since the *Ld*Flabarins tagged at the N- or C-terminus did not co-localize, the localization of native *Ld*Flabarin was unclear. To elucidate this point, we produced a specific anti-*Ld*Flabarin rabbit antiserum. First, C-terminus His6-tagged recombinant *Ld*Flabarin (*Ld*Flabarin-His6) was synthesized in *E. coli* using the pET29b expression vector. After IPTG induction, a 55 kDa band (for a predicted 41.3 kDa, including the His6-tag) was visible in extracts submitted to SDS-PAGE (Fig. 2A, SI) while no band was visible without induction (Fig 2A, SNI). The difference between the predicted and the observed molecular masses may be due to intrinsic migration properties. Surprisingly, under non-denaturing conditions, partially purified *Ld*Flabarin-His6 (Fig 2A, F16) presented several high molecular mass bands (∼240, ∼360, and ∼570 kDa, Fig. 2B). When submitted to a denaturing second dimension electrophoresis, these high molecular mass bands all dissociated to 55 kDa spots (Fig 2C1), all of them being reactive to anti-His6 antiserum (Fig 2C2); thus, the complexes comprised mostly *Ld*Flabarin-His6 proteins devoid of major *E. coli* contaminants, since no other spot was visible by Coomassie staining, although the presence of minor components, eventually responsible for the molecules aggregation, cannot be excluded.

For the rabbit immunization, the recombinant *Ld*Flabarin-His6 was partially purified (Fig 2A, F16). The obtained anti-*Ld*Flabarin antiserum was used to probe western-blots of *L. amazonensis* cell extracts. Native *La*Flabarin was detected as a 50 kDa band (for a predicted 37.8 kDa; Fig. 2D, left panel, lane 1); no band was seen with the preimmune serum (not shown) or an anti-mRed antiserum (Fig 2D, right panel, lane 1). Using *Ld*Flabarin-mRed-expressing cell extracts, the anti-*Ld*Flabarin recognized a main additional band of 90 kDa (Fig. 2D, left panel, lane 2) (for a predicted 63.3 KDa), also detected by the anti-mRed antiserum (Fig 2D, right panel, lane 2). The difference between the predicted and the observed molecular masses is not clear at the moment, and may be due to post-translational modifications or intrinsic migration properties. An additional 75 kDa band was also detected by the anti-*Ld*Flabarin (Fig 2D, left panel, lane 2); however, the intensity of this extra-band, feebly detected by the anti-mRed antiserum (Fig 2D, right panel, lane 2), was variable from an experiment to another, suggesting the existence of a preferential cleavage site within the *Ld*Flabarin-mRed sequence; the protease(s) involved could be partially active in spite of the presence of an anti-protease cocktail during the cell lysis, although it remains possible that this partial proteolysis occurred before lysis.

Probing untransformed *L. amazonensis* cells by indirect immunofluorescence, the anti-*Ld*Flabarin revealed a punctuated labelling along the flagellum (Fig. 2E1 and E2). As with the GFP/mRed fusion proteins, a spot was sometimes observed at the flagellum base (Fig. 2E, lower cell). The flagellum most distal part remained unlabelled (Fig. 2E, both cells); whether the endogenous *La*Flabarin was absent or in too low amount to be detected by the antiserum remains unknown; conversely, the more abundant tagged *Ld*Flabarin-GFP was visible from bottom to tip of the flagellum (Fig. 1B). In conclusion, our data confirmed the flagellar localization of native *La*Flabarin and validated the observations made with the C-terminally tagged *Ld*Flabarin-GFP (Fig. 1B): a free N-terminus is indispensable for *Ld*Flabarin flagellar addressing.

### Ultrastructural localization of *Ld*Flabarin

**Figure 3:**
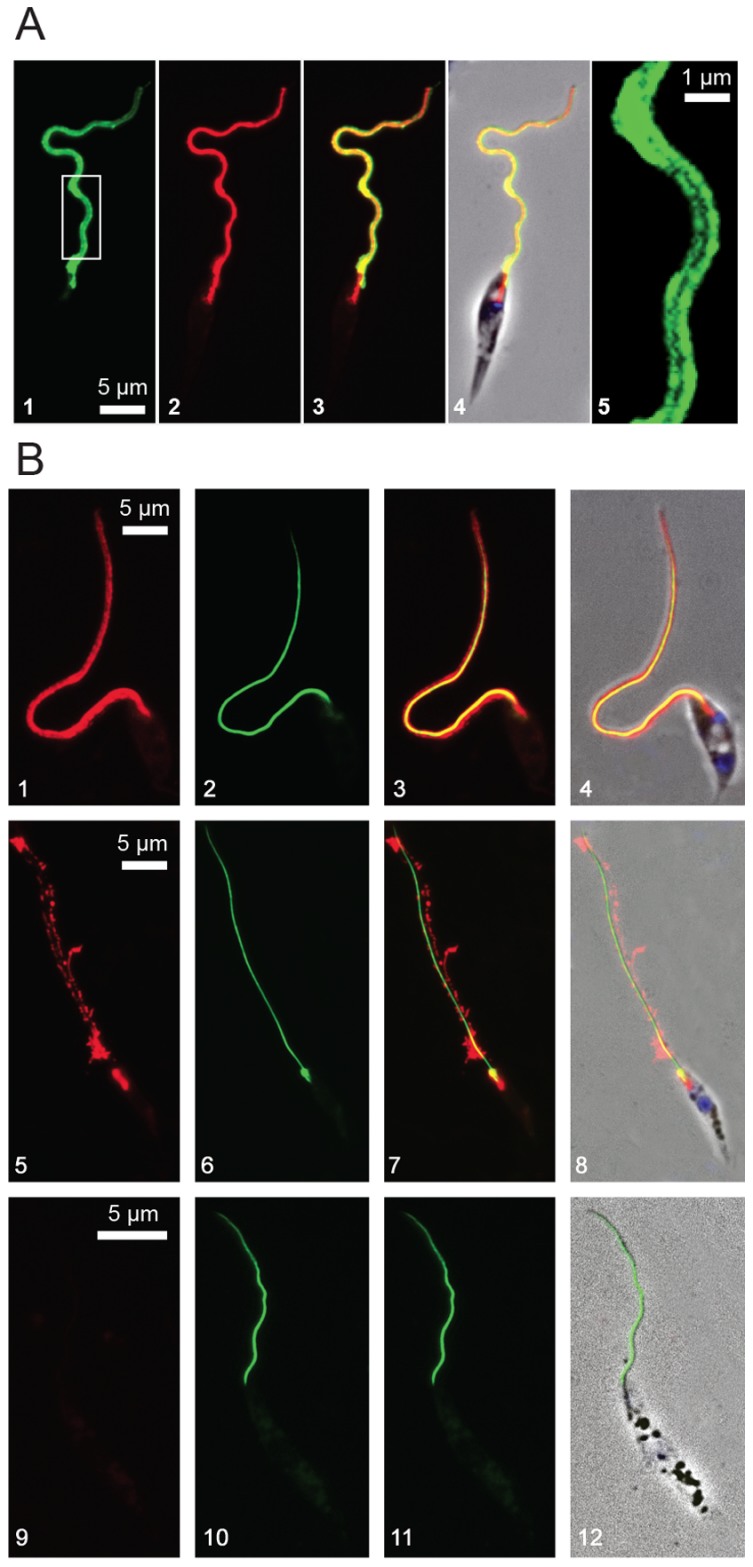
*Ld*Flabarin is not associated with the flagellar cytoskeleton. (A) Comparison of *La*Flabarin/*Ld*Flabarin-mRed localization by immunodetection with rabbit anti-*Ld*Flabarin and mRed fluorescence. *L. amazonensis* BA125 cells expressing *Ld*Flabarin-mRed were treated with anti-*Ld*Flabarin plus Alexa-labelled anti-rabbit IgG. A1, native *La*Flabarin and *Ld*Flabarin-mRed revealed by anti-*Ld*Flabarin plus Alexa-labelled anti-rabbit IgG (green); A2, same cell, *Ld*Flabarin-mRed revealed by mRed fluorescence (red); A3, overlay of A1 and A2; A4, overlay of A1, A2, DAPI staining (blue) and phase contrast images; A5, magnification of the framed area of A1. (B) Co-expression of *Ld*Flabarin-mRed and GFP-*La*PFR2C in *L. amazonensis* BA125. Cells were co-transfected with pNUS *Ld*Flabarin-mRedcD (red, B1) and pNUS GFPnH-*La*PFR2C (green, B2; B3 is an overlay of B1 and B2), stained with DAPI (blue, B4). B5–12, NP-40 treatment. Prior to PFA fixation, cells co-expressing *Ld*Flabarin-mRed and GFP-*La*PFR2C were treated for 5 min at room temperature with 0.0005% (B5–8) and 0.001% (B9–12) NP-40. B1/B5/B9, *Ld*Flabarin-mRed (red); B2/B6/B10, GFP-*La*PRF2C (green); B3/B7/B11, overlay image of B1–2/B5–6/B9–10; and B4/B8/B12, overlay image of B1–2/B5–6/B9–10 plus DAPI staining (blue) and phase contrast.

When observed by indirect immunofluorescence (anti-*Ld*Flabarin plus Alexa-labelled anti-rabbit IgG), the green fluorescence signal of *Ld*Flabarin-mRed (Fig. 3A1) was usually confined to the periphery of the flagellum, while the inside region remained darker (Fig. 3A1 and A5), as expected for a plasma membrane labelling; this pattern was less obvious with *Ld*Flabarin-GFP (Fig. 1B) or the direct visualization of *Ld*Flabarin-mRed fluorescence (Fig. 3A2), which could be attributed to different diffusion characteristics of the fluorophores. For comparison, we used a paraflagellar rod (PFR) marker. PFR is a cytoskeletal structure of trypanosomatid, euglenoid and dinoflagellate flagella [32] that extends inside the flagellum, along the axoneme, from the exit of the flagellar pocket to the tip of the flagellum [33]. Its main constituents are two structurally related proteins, PFR1 and PFR2 [33], [34], which, as other cytoskeleton-associated proteins, remain insoluble after treatment with non-ionic detergents [35], [36]. When expressed as fusion proteins with GFP at their N-terminus, PFR proteins are addressed to the flagellum [37]. Here the *L. amazonensis* PFR2C (one of the tandemly repeated isoforms fused to GFP) was co-expressed with *Ld*Flabarin-mRed in *L. amazonensis*. GFP-*La*PFR2C was visible as a thin flagellar filament (Fig. 3B2), whereas *Ld*Flabarin-mRed extended further inside the flagellar pocket and, in that particular case, was visible as two external red filaments on each side of the green GFP-*La*PFR2C signal (Fig. 3B1, B3 and B4); clearly, *Ld*Flabarin-mRed did not co-localize with GFP-*La*PFR2C.

To investigate the possible association of *Ld*Flabarin with the flagellar cytoskeleton, cells co-expressing GFP-*La*PFR2C and *Ld*Flabarin-mRed were treated with increasing concentrations (0.0005 to 1%) of the non-ionic detergent NP-40 before paraformaldehyde (PFA) fixation (Fig. 3B). From 0.0005% (Fig. 3B5–8) to 0.001% (Fig. 3B9–12) up to 1% NP-40 (not shown), GFP-*La*PFR2C remained associated to the cell ghosts (Fig. 3B6 and B10), while *Ld*Flabarin-mRed was destabilized with as little as 0.0005% NP-40 (Fig. 3B5) and completely solubilized with 0.001% NP-40 (Fig. 3B9), indicating that *Ld*Flabarin is not associated with the flagellar cytoskeleton.

**Figure 4:**
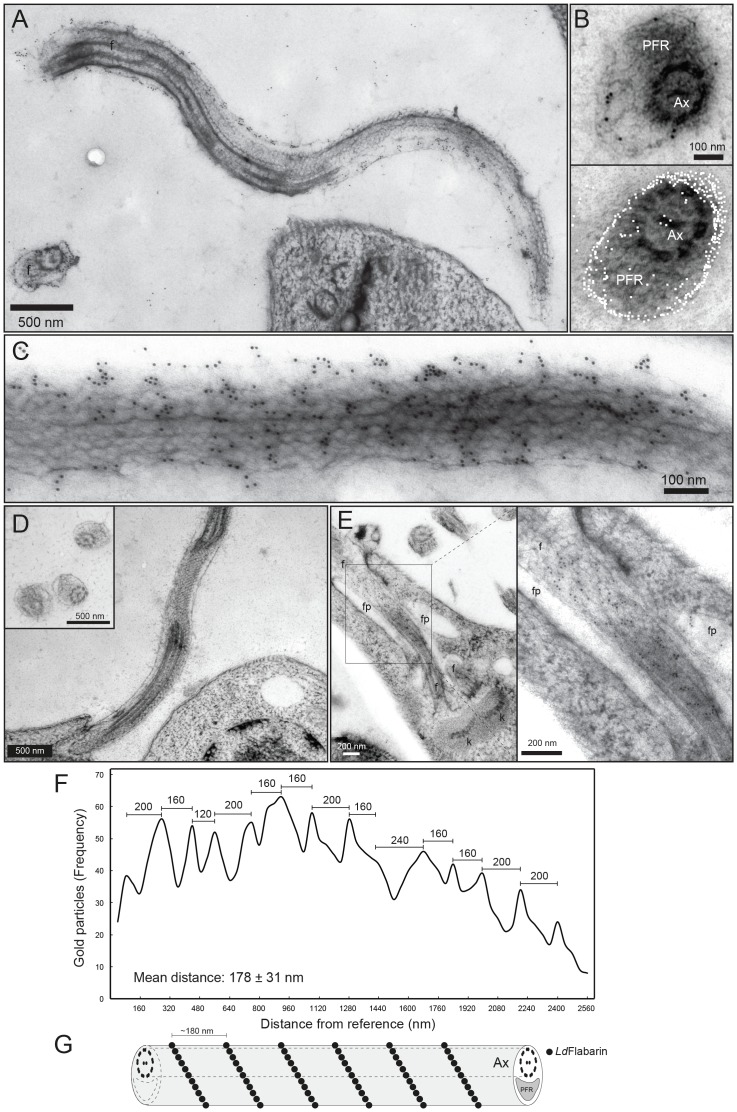
Ultrastructural localization of *Ld*Flabarin. (A) Longitudinal section of a flagellum of *Ld*Flabarin-mRed-expressing *L. amazonensis* using anti-*Ld*Flabarin and an anti-rabbit IgG-gold particle conjugate; black dots represent gold particles; f, flagellum. (B) Upper panel: transverse section of a flagellum of *Ld*Flabarin-mRed-expressing *L. amazonensis* using the anti-Red antiserum. PFR, paraflagellar rod; Ax, axoneme; black dots, gold particles. Lower panel: localization of 383 gold particles (white dots) cumulated from 16 transverse cross-section images. (C) Longitudinal section of a flagellum of *Ld*Flabarin-mRed-expressing *L. amazonensis* (anti-Red antiserum as in B); black dots, gold particles. (D) Same as A and B, except no anti-*Ld*Flabarin antiserum. (E) Vicinity of the flagellar pocket of a *Ld*Flabarin-mRed-expressing *L. amazonensis* cell (anti-Red antiserum as in B); black dots, gold particles; f, flagellum; fp, flagellar pocket; k, kinetoplast. (F) Gold particle frequency along the flagellum as a function of distance from the reference point (first gold particle counted); distance measurements (4546 gold particles from 50 different flagella) were performed using ImageJ software. (G) Schematic representation of *Ld*Flabarin organization in the flagellum: black circles indicate *Ld*Flabarin; Ax and PFR are also represented.

Then we performed immuno-electron microscopy on non-transfected and *Ld*Flabarin-mRed-expressing *L. amazonensis* cells using anti-*Ld*Flabarin or a rabbit anti-mRed antibody plus an anti-rabbit IgG-gold particles conjugate. The gold particles (black dots) localized to the flagellar plasma membrane (Fig. 4A and B upper panel) in longitudinal cross-sections of *L. amazonensis* cells. The specificity of the labelling was ascertained by the absence of any black dot when the anti-*Ld*Flabarin primary antiserum was omitted (Fig 4D). A cumulative transverse cross-sectional image was generated (Fig. 4B, lower panel; artificial color: white dots), and *Ld*Flabarin-mRed appeared both randomly distributed on the surface of the flagellum and randomly oriented with respect to the axoneme or the PFR. Conversely, in longitudinal sections, the black dots appeared clustered in small bundles, obliquely aligned and separated by a mean distance of 178±31 nm (Fig. 4C and F), compatible with a helicoidal arrangement around the flagellum (Fig. 4G). At the base of the flagellum, where *Ld*Flabarin-mRed/GFP occasionnally accumulated (Fig. 1B and 2E1), dots exclusively localized to the flagellum, but not to the flagellar pocket membrane or lumen (Fig. 4E).

**Figure 5:**
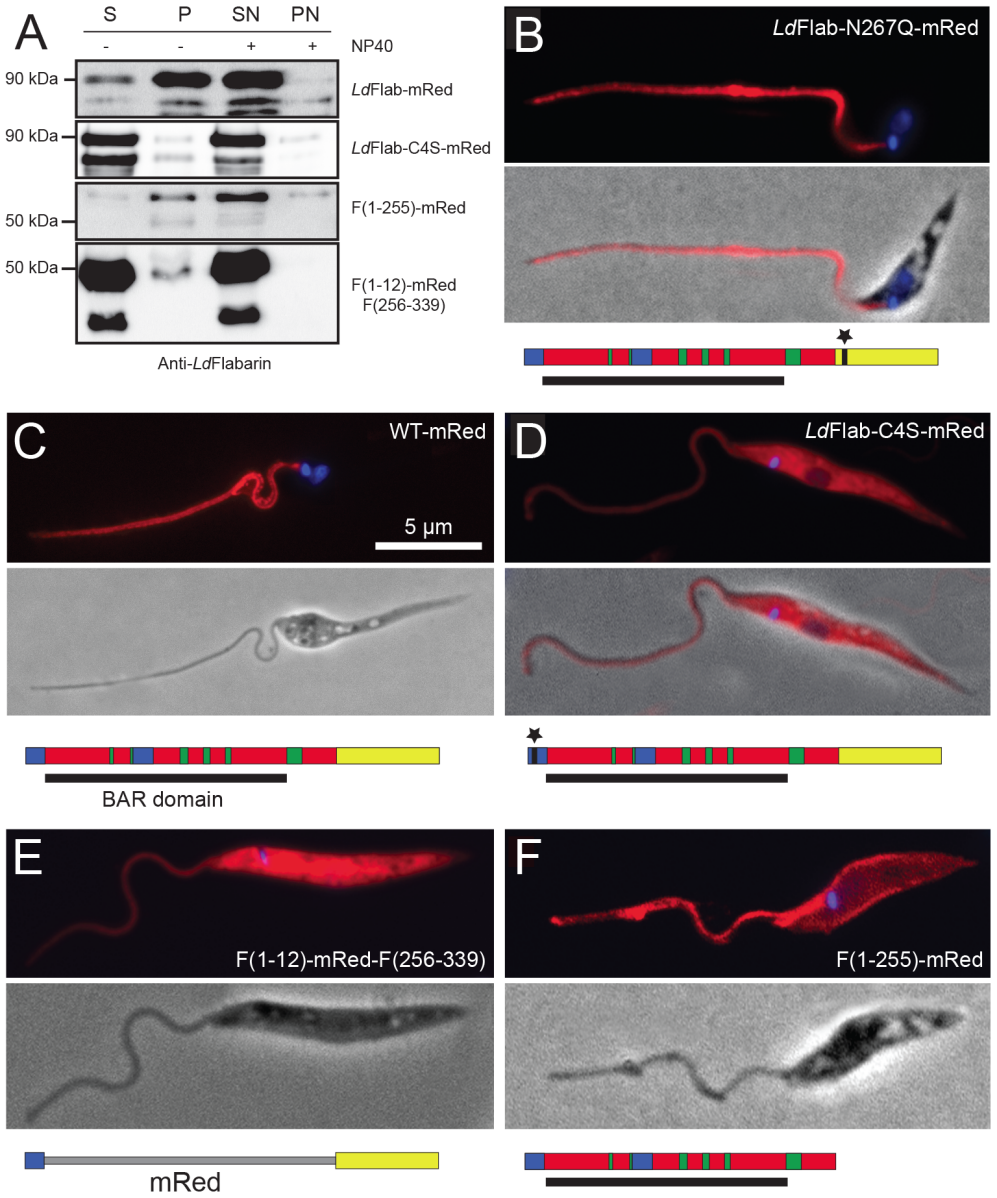
Localization of *Ld*Flabarin-mRed deletion mutants and chimeras. (A). *L. amazonensis* cells expressing *Ld*Flabarin-mRed, *Ld*Flabarin/C4S-mRed, F(1–255)-mRed and F(1–12)-mRed-F(256–339) were fractionated into soluble and membrane fractions by 100 000 g centrifugation in presence and absence of 0.5% NP-40; equivalent of 7.5 10^6^ cells supernatants (S and SN, N for NP-40) and pellets (P and PN) were submitted to SDS-PAGE and western blotting with anti-*Ld*Flabarin as in Fig 2D. (B–E) *L. amazonensis* expressing red fluorescent proteins were fixed, DAPI stained for nuclear (not always visible) and kinetoplast DNAs coloration (blue), and observed under a fluorescence microscope. Constructs are schematically represented by a multicolored bar with the same color codes as in Figure S2; the BAR domain is schematized by a black bar under the constructs. (B) *Ld*Flabarin/N267Q-mRed. (C) *Ld*Flabarin-mRed (1–339). (D) *Ld*Flabarin/C4S-mRed. (E) F(1–12)-Red-F(256–339). (F) F(1–255)-mRed. Bars correspond to 5 µm.

Consistently, after cell fractionation, the 90 kDa band of *Ld*Flabarin-mRed was mainly associated with the membrane fraction (100 000 g pellet) (Fig 5A lane P) compared to the soluble fraction (Fig 5A lane S) while it translocated to the soluble fraction in the presence of detergent (Fig 5A lane SN versus PN). The additional 75 kDa band, which behaved like the 90 kDa band in fractionation experiments, was present in variable amounts, depending of the lysate (Fig 5A) which confirmed it may be the result of a limited proteolysis. The presence of a small amount of *Ld*Flabarin-mRed in the 100 000 g supernatants maybe reflected the occasional protein accumulation observed at the base of the flagellum, which did not seem to be associated to membranes according to the electron microscopy images (Fig. 4E).

Taken together, these data demonstrate a plasma membrane localization and point to a structural function for *Ld*Flabarin.

### Predicted consensus motifs of *Ld*Flabarin

Trypanosomatid Flabarin sequences were analyzed for the presence of consensus motifs and potential post-translational modification sites, and their conservation among orthologues (Table S1).

Many *Leishmania* membrane proteins are heavily and variably glycosylated [38], [39]. *Ld*Flabarin has a potential N-glycosylation site at asparagine 267 (N267), conserved in all *Leishmania* Flabarins but absent in *Trypanosoma* Flabarins (their sequence being shorter; Fig. S1B). Inactivation of this site by replacement of the asparagine 267 by a glutamine (*Ld*Flabarin/N267Q-mRed) did not affect flagellar localization (Fig. 5B) (nor cell viability).

Some membrane proteins require acylation (palmitoylation) of N- or C- terminal cysteine residues for membrane anchoring [40]. Using the CSS-Palm software [41], we found a potential palmitoylation site in the *Ld*Flabarin N-terminus (amino acids 1–7, MPLCASI) with a palmitoylable cysteine at position 4. This site is conserved in *Leishmania* and *Trypanosoma* Flabarins, with the exception of *T. vivax* (Table S1). The cysteine 4 was replaced by a serine (*Ld*Flabarin/C4S-mRed); as a consequence, the potential palmitoylation site was destroyed. Compared to the flagellar wild-type *Ld*Flabarin-mRed (Fig 5C), the fluorescence was diffuse throughout the cell body (Fig. 5D). Cell fractionation showed that the mutant protein *Ld*Flabatin/C4S-mRed was not associated with the membrane fraction (Fig 5A, lane P) but remained soluble in the 100 000 g supernatant (Fig 5A, lane S). Thus, the cysteine 4 was indispensable for the membrane localization as for the flagellar addressing of *Ld*Flabarin.

### Structural domains of *Ld*Flabarin

The amino acid sequence of *Ld*Flabarin was analyzed by several online programs for structure predictions. As summarized in Fig. S2, *Ld*Flabarin comprise (i) a short N-terminal β-strand (S1, aa 1–15), (ii) a putative BAR domain (aa 16/22 to 199/218, limits depending on the prediction program) with 6 α-helices (H1–6), a β-strand (S2) and 5 loops (L1–5), (iii) a “linker” consisting of one loop (L6) and one α-helix (H7), and (iv) a C-terminal disordered domain (D, aa 256–339). The D domain may overlap with loop L6/helix H7 and may comprise an additional α-helix (H8) (not shown). Three amphiphilic helix-rich regions (AHR) were detected within the BAR domain (AHR-1, aa 19–123; AHR-2, aa 202–221) and the C-terminus with a lower probability (AHR-3, aa 291–335). Three potential dimerization regions were predicted on each side of the BAR domain. Although this model will require validation by the 3D-structure determination of *Ld*Flabarin, it provides useful insights on the properties of the protein.

#### The BAR domain

**Figure 6:**
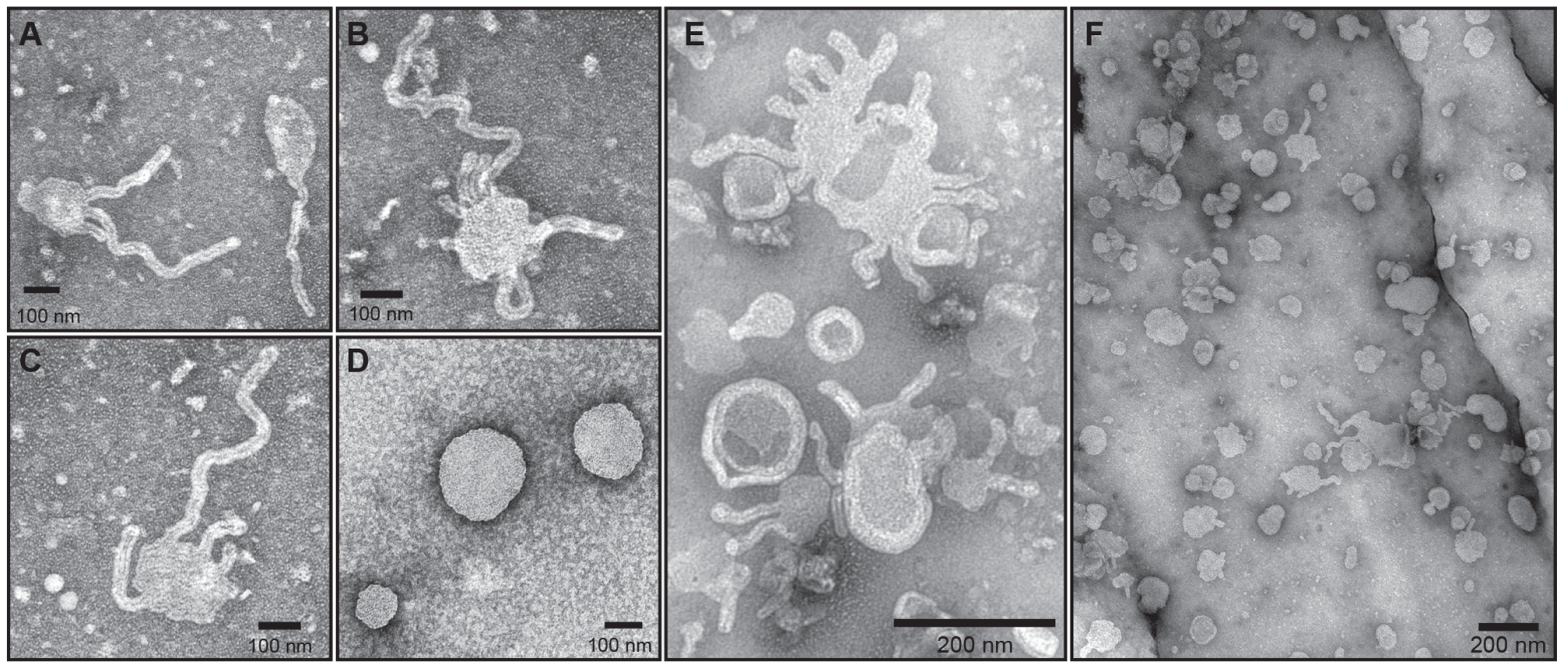
*Ld*Flabarin binds and tubulates liposomes in vitro. Electron micrographs of liposomes incubated with purified recombinant *Ld*Flabarin-His6 (A–C, F) with *Ld*Flabarin without His-tag (E) or with BSA (D).

Proteins of the BAR domain superfamily bind and tubulate liposomes in vitro [42], [43] and anchor proteins to membranes in vivo. To test the prediction of the presence of a functional BAR domain within *Ld*Flabarin, we produced liposomes and examined their morphology by electron microscopy in the presence and absence of *Ld*Flabarin. Incubation with purified *Ld*Flabarin-His6 resulted in deformations of the liposomes that exhibited tubules of similar diameter (15.9±1.7 nm, mean of 92 measurements) but various length (Fig. 6A–C and F). Tubule length varied with protein concentration and incubation time but diameter was constant and there was no branching. By contrast, incubation without *Ld*Flabarin-His6 showed no liposome deformation/tubulation (Fig. 6D). To rule out the possibility that the His6-tag was responsible for the tubulation, it was removed by thrombin digestion, the protein was further purified and similar tubulation occurred (Fig 6E). Thus, like other BAR domain–containing proteins, *Ld*Flabarin was able to bind lipids and induce membrane curvature in vitro.

Concerned with its role in vivo, we replaced *Ld*Flabarin BAR domain by the mRed protein and expressed the chimera F(1–12)-mRed-F(256–339) in *L. amazonensis*. The protein localized diffusely to the entire cell body (Fig. 5E); cell fractionation revealed that it was soluble, absent from the 100 000 g pellet (Fig 5A, lane P) but present in the supernatant (Fig 5A, lane S) (same with detergent, Fig 5A, lanes SN and PN). Thus the putative BAR domain was necessary for *Ld*Flabarin flagellar addressing.

However, the domain was not sufficient for *Ld*Flabarin flagellar addressing, as the protein F(1–255)-mRed, obtained after deletion the tail (aa 256–339), localized mainly to membranes (Fig 5F) and could be found in the 100 000 g pellet (Fig 5A, lane P) but not in the supernatant (Fig 5A, lane S) except in the presence of detergent (Fig 5A lane SN).

In conclusion, *Ld*Flabarin is a flagellar protein; its flagellar addressing depends on three determinants: an N-Terminal potential acylation (palmitoylation) site and a BAR domain, which direct the protein to plasma membranes, and a C-terminal domain which directs the membrane protein to the flagellum.

## Discussion

### Flabarins are not Arfaptin homologues and are unique to eukaryotes

We report the characterization of Flabarin, a novel *Leishmania* flagellar protein. It was identified based on its sequence homology to *Hs*Arfaptin-1. *Hs*Arfaptin-1/-2 localize to the trans-Golgi network (TGN) [25]; they interact with the small G proteins *Hs*Rac-1 [28], *Hs*ARF-1, -3, -6 [25], [44] and participate in the assembly of trafficking vesicles together with *Hs*ARF-1 [45] or *Hs*ARL-1 [46], [47]. On the other hand, *Leishmania* Flabarins are flagellar proteins; their ultrastructural localization and arrangement make it unlikely that they participate in intracellular trafficking. Besides, a close look at the sequence alignments (Fig. S1A) reveals that Flabarins and Arfaptins share identity only in the BAR domains which are located in the C-terminus of *Hs*Arfaptin-1 and in the N-terminus of *Lm*Flabarin. Flabarins belong to only one orthologous group, the Group OG5_148786 [30], which exists only in trypanosomatids. Thus, these proteins are certainly not functional homologues.

### Flabarins are flagellar proteins

The flagellum is a complex structure, comprising notably a lumen, a plasma membrane, an axoneme, and in the case of trypanosomatids, an additional structure called the PFR (paraflagellar rod). Flagellar targeting consists probably in several different mechanisms, for every protein must be targeted to its specific location and there are several hundreds of them. These mechanisms are being progressively deciphered [48] and reveal complex. Sequence motifs have been found to be involved in a specific flagellar targeting; for example, a simple HLA C-terminal motif is necessary, but not sufficient, for the proteins PFRA and ARP to reach their location in the *T. brucei* PFR structure [37], [49], while another N-Terminal motif is necessary and sufficient to address the protein ADK-A to the same structure [50]. Concerning flagellar membrane proteins, two main mechanisms have been documented. The first one concerns the *Leishmania* glucose transporter, where particular internal epitopes and amino-acids are essential [51], [52]. The second category of proteins must be myristoylated and palmitoylated at their N-termini for reaching the flagellar membrane; it is the case of the *T. cruzi* FCaBP [53], [54], of the *T. brucei* Calflagins [55] and of the *Leishmania* HASPB [56] and SMP-1 [57], [58].

For *Ld*Flabarin, flagellar membrane targeting should involve another mechanism: there is no myristoylation site, only a potentially palmitoylable site at the N-terminus, there is a BAR domain, which does not exist in the other cases, and the C-terminus provides the flagellar specificity. In preliminary experiments, we expressed the dominant-negative mutant *Ld*ARL-1/T34N, which blocks TGN vesicular trafficking [59]: we observed that the flagellar localization of *Ld*Flabarin-mRed was not modified (not shown), which raises questions about the trafficking pathway used by the protein for reaching its destination. It is being recognized that proteins do not diffuse freely from the membrane of the cell body to the flagellar membrane, and a diffusion barrier has been repeatedly invoked recently for controlling access to the flagellum (or cilium) [60]–[63]. More work is needed to address these problems.

### Flabarins are original BAR domain proteins

First found in amphiphysin, a synaptic vesicle protein [64], later in the yeast Rvs161 protein [18], BAR domains represent an expanding family [14] belonging to a variety of multi-domain proteins from eukaryotes, including the protozoans *T. brucei* [65] and *Leishmania* (this work). Several sub-families have been defined, including the archetype Arfaptin BAR domain, the N-, F-, I- and SNX-BAR domains [17], [19], [66]. The basic BAR domain consists of three α-helices folded onto each other which dimerize to form a banana-shaped structure that binds to lipid membrane, recognizes and induces curvature [67]. Additional structures help the BAR domains in membrane anchoring, e.g. the N-terminal amphipathic α-helix of the N-BAR domain of endophilin-1 [68], while neighbouring domains (e.g., PH domains) provide the organelle membrane specificity [17].

The *Ld*Flabarin BAR domain and structure have been predicted by online programs: several α-helices are recognizable between amino acids 16/22 and 218, but the real structure remains to be determined; crystallization attempts are actively pursued. *Ld*Flabarin binds to, deforms and induces the tubulation of lipid membranes in vitro (Fig. 6) like other BAR domains proteins do. Moreover, our deletion studies uncovered two distinctive features of the *Ld*Flabarin BAR domain. First, the BAR domain is necessary, but not sufficient, for membrane binding in vivo, which requires the presence of cysteine 4, a potentially palmitoylated amino acid, and suggests that palmitate could play the same membrane-anchoring role as the N-terminal amphipathic α-helix of N-BAR domains; to our knowledge, it is the first BAR domain of the sort. Second, the flagellar addressing, which depends on the C-terminal region, makes *Ld*Flabarin the first BAR domain protein localized to a flagellum.

BAR domains are dimerization domains. Dimerization regions have been predicted for *Ld*Flabarin (http://www.ncbi.nlm.nih.gov/Structure/cdd/cdd.shtml [69]). Although our data need confirmation, they suggest that native *Ld*Flabarin auto-associates to form oligomers of discrete sizes (Fig. 2B–C); the ultrastructural helicoidal arrangement around the flagellum would be consistent with such a controlled oligomerization (Fig. 4). In this perspective, we are planning to perform in vitro and in vivo interaction studies with recombinant truncated proteins.

### Potential role of Flabarins

Many BAR domain proteins are involved in the biogenesis of membrane vesicles, by protrusion (intracellular traffic) and invagination (endocytosis), depending on the concave or convex curvature they induce or recognize. It is difficult to envision such processes at the flagellum surface because in *Leishmania*, vesicular endocytosis/exocytosis occur exclusively inside the flagellar pocket [70]. Given its helicoidal arrangement along the flagellum, *Ld*Flabarin could have a morphogenetic function. The nature of the BAR domain (i.e., the banana-shaped dimer) is known to determine the curvature of the membrane to which it binds. Thus, “F-BAR domains typically induce wider membrane tubules compared with the ones induced by BAR/N-BAR domains” [71]; however, there is a large difference in diameter between the *Ld*Flabarin-induced tubules in vitro (15.9±1.7 nm) and the flagellum (308±38 nm). *Ld*Flabarin alone could not generate the flagellum as a large tubule but may help stabilizing its structural complex.

Gene disruption/replacement experiments are in progress, which will hopefully provide some understanding of *Ld*Flabarin role and its involvement in *Leishmania* flagellar assembly, structure or functioning.

## Materials and Methods

### Cell culture and transfection

*Leishmania amazonensis* (MHOM/BR/1987/BA125; MHOM/BR/1987/BA276) and *L. donovani* (MHOM/ET/1967/Hu3:LV9) promastigotes were cultured at 24°C in AM medium with 7.5% FCS [21], [72]. Electroporations were done in duplicate with 50 µg plasmid, and stable transfectants selected by addition of 50 µg/ml hygromycin (Euromedex) or 10 µg/ml blasticidin (InvivoGen) 24 h later [21].

### DNA technology

Conventional methods were used for DNA extractions [73]. Restriction and modification enzymes were from New England Biolabs, oligonucleotides from Eurofins MWG Operon. DNA fragments were amplified with Phusion DNA polymerase (Finnzymes) and cloned into the pUC-18 or pMOS vectors (SureClone Ligation Kit or Blunt Ended PCR Cloning Kit, GE Healthcare) using the *E. coli* strain XL1-Blue (Stratagene). Inserts were sequenced, analyzed with DNA Strider 1.4f14 [74], and recloned between Nde I and Kpn I sites (unless otherwise mentioned) into pNUS-GFPnH/cH (conferring hygromycin resistance) or pNUS-mRednD/cD (conferring blasticidin resistance) vectors [75], [76], allowing expression in *Leishmania* of proteins fused at their N/C-terminus either to the Green Fluorescent Protein (GFP) or the monomeric Red Fluorescent Protein (mRFP [77], named here mRed). All constructs were sequenced prior to transfection.

### Plasmid constructions

The ORF was PCR amplified from *L. donovani* LV9 genomic DNA with oligonucleotides (designed from the *L. major* Friedlin genome [78]) G165(gatcagatctcatATGCCGCTCTGCGCCAGCATC)/G166(gatcggtaccTCACTCATCGTTGTTTGCGTC) or G165/G167(gatcggtaccCTCATCGTTGTTTGCGTCAAC) and cloned into pNUS-mRedcD (generating pNUS-*Ld*Flabarin-mRedcD) or pNUS-mRednD (generating pNUS-mRednD-*Ld*Flabarin), respectively.

For the *Ld*Flabarin/N267Q mutant, the codon AAC (799–801) was mutated to cAg to replace N267 with Q. First, two overlapping fragments were amplified from the *Ld*Flabarin ORF with oligonucleotides G390(gatcaagcttcatATGCCGCTCTGCGCCAGCATC)/G419(GCGGTGGCTGTTcTgCGATGTGACATC) and G418(GATGTCACATCGcAgAACAGCCACCGC)/G391(gatcggatccggtaccCTCATCGTTGTTTGCGTCAAC). Then, the fragments were annealed, *Ld*Flabarin/N267Q amplified with the oligonucleotides G390/G391 and cloned into pNUS-mRedcD (generating pNUS-*Ld*Flabarin/N267Q-mRedcD).

The *Ld*Flabarin/C4S mutant was obtained by amplification from the *Ld*Flabarin ORF with oligonucleotides G436(gatcaagcttcatATGCCGCTCTcCGCCAGCATC (with a c mutation at position 11 to replace C4 with S)/G391 and cloned into pNUS-mRedcD (generating pNUS-*Ld*Flabarin/C4S-mRedcD).

The F(1–255)-mRed mutant was amplified from the *Ld*Flabarin ORF with the oligonucleotides G165/G380(ctcggtaccCGACGCCTCGTTCTTGCGCTG) and cloned into the pNUS-mRedcD vector between Nde I and Kpn I.

The chimera F(1–12)-mRed-F(256–339) was constructed in three steps as follows. First, the C-terminal fragment F(256–339) was amplified from the *Ld*Flabarin ORF with the oligonucleotides G381/G441(gatcggatccggtaccTCACTCATCGTTGTTTGCGTCAAC) and cloned between Nde I and Kpn I sites of pNUS-mRednD (thus replacing the mRed ORF), to generate the vector pNUS-F(255–339). Second, for the N-terminal fragment F1–12 (nt 1–36), 5 µg complementary oligonucleotides G344B(tatgCCGCTCTGCGCCAGCATCCCCGCGACGGTCGACggtac) and G345B(cGTCGACCGTCGCGGGGATGCTGGCGCAGAGCGGca) were boiled together in 40 mM Tris/HCl pH 7.5, 20 mM MgCl2, 50 mM NaCl, 5 mM DTT for 1 min and cooled slowly to room temperature for about 2 h; the annealed fragment was then cloned into pNUS-mRedcD to generate the vector pNUS-F(1–12)-mRedcD. Third, the F(1–12)-mRed fragment was amplified from the pNUS-F(1–12)-mRedcD with the oligonucleotides G390/G440(gatccatatgGGCGCCGGTGGAGTGGCGGCC), cloned (and oriented) into the Nde I site of pNUS-F(255–339), to generate the vector pNUS-F(1–12)-mRed-F(255–339).

*La*PFR2C (Paraflagellar Rod protein 2C) was amplified from *L. amazonensis* BA276 genomic DNA with the oligonucleotides G308B(ctcggtaccagatctcatATGAGCATCGCTGCGGACATGGCGTACCC)/G309(ctcggtaccagatctCTACTCGGTGATCTGTTGCA), as designed from the *L. mexicana* sequence (GenBank U45884) [34]. The *La*PFR-2C ORF (submitted to GenBank, accession number JN874564) comprised 1797 bp/591 amino acids (98%/98.7% identity to *Lmx*PFR2C, respectively [34]) with 36 nucleotides/8 amino acids differences. The ORF was cloned into the Acc65 I site of the pNUS-GFPnH vector (pNUS-GFPnH-*La*PFR2C).

*Ld*Centrin [31] (GenBank AF406767) was amplified from *L. donovani* LV9 genomic DNA with oligonucleotides G446(gatccatATGGCTGCGCTGACGGATGAACA)/G447(gatcggtaccCTTTCCACGCATGTGCAGCA) and cloned into pNUS-GFPcH (pNUS-*Ld*Centrin-GFPcH); its sequence was 100% identical to the GenBank sequence.

### Recombinant *Ld*Flabarin-His6, anti-*Ld*Flabarin rabbit antiserum

The *Ld*Flabarin ORF was excised from the pNUS-*Ld*Flabarin-mRedcD and transferred to the bacterial expression vector pET-29b (Novagen) (pET29b-*Ld*Flabarin-His6) for expression in the *E. coli* strain BL21(DE3). The transformed bacteria were grown at 36°C in LB broth containing 100 µg/ml kanamycin. When cultures reached an OD at 600 nm of 0.6, isopropyl-β-D-thiogalactopyranoside (IPTG) was added to a concentration of 0.4 mM to induce expression of *Ld*Flabarin-His6. Bacteria were harvested 3–4 h later.

Recombinant *Ld*Flabarin-His6 was purified by fast protein liquid chromatography (ÄKTA Purifier system) at 4°C. After IPTG induction, bacteria were resuspended in 10 ml binding buffer (5 mM imidazole in 10 mM Tris pH 8, 300 mM NaCl) and sonicated (3×90 s pulses interrupted with cooling on ice). After elimination of cell debris by centrifugation at 25000 g, the supernatant was applied to a nickel-chelated agarose affinity column (Qiagen). After extensive washes with binding buffer, the protein was eluted with an imidazole gradient (5–1000 mM) and 2 ml fractions were collected. The protein was eluted with about 150 mM imidazole (Fraction 16: F16). Fractions were analyzed by SDS-PAGE. The recombinant protein was concentrated to 1 mg/ml in Hepes 50 mM pH 7.5, 150 mM KCl, glycerol 10% using Vivaspin ultrafiltration device (Vivascience Sartorius, cutoff 10000 Da) and stored at 4°C. Protein concentration was determined by UV spectra and by the Bradford method [79].

Four 5-µg aliquots of *Ld*Flabarin-His6 were injected every other week to a female NZW rabbit (Charles River Laboratories) at the IRD animal facility. The first injection was done with 50% complete Freund adjuvant, the last 3 with incomplete Freund adjuvant). Each time, ten (0.1 ml) aliquots of the homogenate were injected into the right side of the rabbit. The reactivity of the antiserum was tested 10 days after the third injection by Western blot. The final anti-*Ld*Flabarin antiserum was collected 10 days after the fourth injection.

### Cell lysis, cell fractionation and Western blotting

*Leishmania* total protein extraction was done as described [21]. For cell fractionation, 100 ml log phase cells (1.5 10^7^ cells/ml) were washed twice with PBS, resuspended in 1 ml hypotonic Buffer I (10 mM Hepes pH 7.4, 1 mM DTT, 2 mM EDTA, 1/100 dilution of Sigma P8215 protease inhibitors cocktail) and incubated for 10 min in ice. Cells were mechanically broken by 20 passages through a 26GX1/2″ gauge. After addition of cold 3.5 ml Buffer II (same as Buffer I except 50 mM Hepes pH 7.4 instead of 10 mM)±0.5% NP-40, the lysates were cleared by 1500 g 15 min centrifugation and the supernatants further centrifuged at 100 000 g for 1 h at 4°C (SW55Ti rotor, Beckman Coulter Optima LE-80K centrifuge). The last supernatants represented the soluble fractions and the pellets, resuspended in 4.5 ml Buffer II±0.5% NP-40, the membrane fractions.

Western blotting was done as described [21] and revelation as in [59]; anti-*Ld*Flabarin (1∶2000 dilution) was additioned with BL-21 *E. coli* homogenate for eliminating an eventual reactivity of the rabbit serum with bacterial contaminants of the purified recombinant *Ld*Flabarin.

### Fluorescence microscopy

Fluorescence microscopy was done as described [21]. Cells were spread onto poly-L-lysine-treated coverslips and fixed with 4% PFA for 20 min at room temperature. Coverslips were washed 3×5 min with PBS and DNA was stained with DAPI (10 µg/ml) during the last 5-min wash before mounting on microscope slides with Mowiol. For indirect immunofluorescence, coverslips were incubated for 2 h with anti-*Ld*Flabarin (1∶1000 dilution), and for 2 h with 8 µg/ml goat anti-rabbit IgG conjugated to Alexa Fluor 488 (Molecular Probes). Observations were done with an Axioplan 2 Zeiss fluorescence microscope and a 100X oil lens. Images were acquired with a Princeton Instruments or Photometrics CoolSnap HQ camera and analyzed with Metaview (Universal Imaging) and Adobe Photoshop. Acquisition times: phase contrast, 100 ms; DAPI, 50 ms; red and green channels, 50–500 ms, depending on the fluorescence level to avoid saturation.

### Freezing and freeze substitution for ultrastructural studies

*Leishmania* pellets (0.5–1 10^9^ cells) were washed three times in PBS buffer, incubated with 2% dextran in PBS buffer for 1 h and placed on the surface of a Formvar-coated copper electron microscopy grid (400 mesh). Each loop was quickly submersed in precooled liquid propane and kept at −180°C in liquid nitrogen. The loops were incubated in 4% osmium tetroxide in dry acetone at −82°C for 48 h (substitution fixation), gradually warmed to room temperature, and washed three times in dry acetone. Specimens were stained for 1 h with 1% uranyl acetate in acetone at 4°C in the dark. After another rinse in dry acetone, the loops were infiltrated progressively with araldite (epoxy resin; Fluka). Ultrathin sections were contrasted with lead citrate.

### Immunogold electron microscopy

*Leishmania* cells were cryofixed in 2% dextran as above and freeze substituted with acetone plus 0.1% glutaraldehyde for 3 days at −82°C. Samples were rinsed with acetone at −20°C and embedded progressively at −20°C in LR Gold resin (EMS). Resin polymerization was carried out at −20°C for 3 days under UV illumination, after which ultrathin LR Gold sections were collected on Formvar-coated nickel grids. Sections were incubated at room temperature with 1 mg/ml glycine for 5 min, with FCS (1∶20) for 5 min, with anti-Red antiserum (1∶250) (MOLBIO ML75489) or anti-*Ld*Flabarin (1∶2000) for 45 min and with an anti-rabbit/10 nm gold particles conjugate (1∶500) (BioCell) for 45 min. The sections were rinsed with distilled water and contrasted for 5 min with 2% uranyl acetate in water followed by 1% lead citrate for 1 min. Specimens were observed with a HITACHI 7650 electron microscope (Electronic Imaging Pole of Bordeaux Imaging Center).

### Liposome preparation and in vitro tubulation assays

Phosphatidylcholine (DOPC) and phosphatidylethanolamine (DOPE) (ratio: 70/30; 10 mg/ml in chloroform; Avanti Polar Lipids) were dried under a stream of argon and kept under vacuum for at least 2 h. The lipids were then redissolved in Hepes 50 mM pH 7.5, KCl 150 mM, glycerol 10% to a final concentration of 1 mg/ml, gently vortexed, subjected to seven freeze–thaw cycles, and immediately extruded 21 times through a 50- or 100-nm polycarbonate membrane (Avanti Polar Lipids). The homogeneity of the liposome preparations was tested using dynamic light scattering (DLS). For tubulation assays, 10 µl of liposomes were incubated with 10 µl of recombinant *Ld*Flabarin-His6 (1 mg/ml = 24 µM) or *Ld*Flabarin without Histidine-Tag (repurified after thrombin digestion) for 2 h at room temperature. Samples were applied for 15 min to Formvar/carbon-coated copper grids, washed with distilled water and stained with 2% uranyl acetate in water for 1 min. Specimens were examined on a HITACHI 7650 electron microscope as above.

## Supporting Information

Figure S1 **A: Sequence alignment between** ***H. sapiens*** **Arfaptin-1 and** ***L. major*** **Flabarin.** Alignment of *Hs*Arfaptin-1 (Genbank U52521) and *Lm*Flabarin (LmjF.27.1730) obtained from GeneDB [GeneDB-Blast-Lmajor, 2013 #13595]. Score  = 84 (34.6 bits), Expect  = 0.033, P = 0.032. Identities  = 54/213 (25%), Positives  = 88/213 (41%). Identical amino acids are highlighted in black, similar amino acids (apolar, polar) in grey. **B: Sequence alignment of** ***Ld*** **Flabarin and its orthologues.** Clustal W (1.83) (http://www.ch.embnet.org/software/ClustalW.html) alignment of *Ld*Flabarin and its orthologues (Group OG5_148786, http://orthomcl.org): *L. infantum* (*Lin*J.27.1630), 99,7% id.; *L. major* (*Lmj*F.27.1730), 90,3% id.; *L. mexicana* (*Lmx*M.27.1730), 88,2% id.; *L. braziliensis* (*Lbr*M.27.1860), 73,5% id.; *Trypanosoma cruzi* (*Tc*CLB.506125.20 indicated by ♯, and *Tc*CLB.504153.30), 20,3 and 19,8% id., respectively; *T. vivax* (*Tv*Y486_0013090), 19,2% id.; *T. congolense* (*Tc*IL3000.11.2210.1), 18,3% id.; *T. brucei* (*Tb*927.11.2410, formerly *Tb*11.22.0001), 18% id. Two other orthologues, *Tb*427tmp.22.0001 (from another *T. brucei* strain) and *Tbg*972.11.2660 (*T. gambiense*), were not included because their amino acid sequences are identical to *Tb*927.11.2410. Identical amino acids are highlighted in black; *, and: correspond to “identity”, “semi-conservative substitution” and “conservative substitution”, respectively.
(TIF)

Figure S2 **Analysis of structural domains and motifs of** ***Ld*** **Flabarin.** Schematic representation of *Ld*Flabarin structural domain predictions by NCBI-Blast (http://www.ncbi.nlm.nih.gov/blast/Blast.cgi), secondary structure predictions by PredictProtein (http://www.predictprotein.org/) and Disopred (http://bioinf.cs.ucl.ac.uk/disopred/), and potential amphiphilic helices by Heliquest (http://heliquest.ipmc.cnrs.fr). Amino acids are numbered starting with the first methionine. NB: (i) the α-helix H7 (227–256; PredictProtein) is considered as belonging to the disordered region (D) by Disopred and (ii) there may be a α-helix in the middle of the disordered region (PredictProtein). PredictProtein/Disopred: red is for α-helix (H), blue for β-strand (S), green for loop (L), and yellow for disordered (D); solid lines limited by solid triangles correspond to potential dimerization domains. Heliquest: AHR, amphiphilic α-helix region. The four selected 18-aa windows show α-helices as viewed from above: for a comparison, the first on the left (M1-R18) is not amphiphilic, the other three are; non-polar amino acids are yellow, grey, and green; polar amino acids are blue for cationic, red for anionic, and pink and purple for neutral.
(TIF)

Table S1 **Potential modification sites of Flabarins and conservation between species.**
(DOC)
