# Supplementary figures and images for: LdFlabarin, a New BAR Domain Membrane Protein of Leishmania Flagellum
Source: PLoS One. 2013 Sep 27;8(9):e76380. doi: 10.1371/journal.pone.0076380 (PMC3785460; doi:10.1371/journal.pone.0076380)

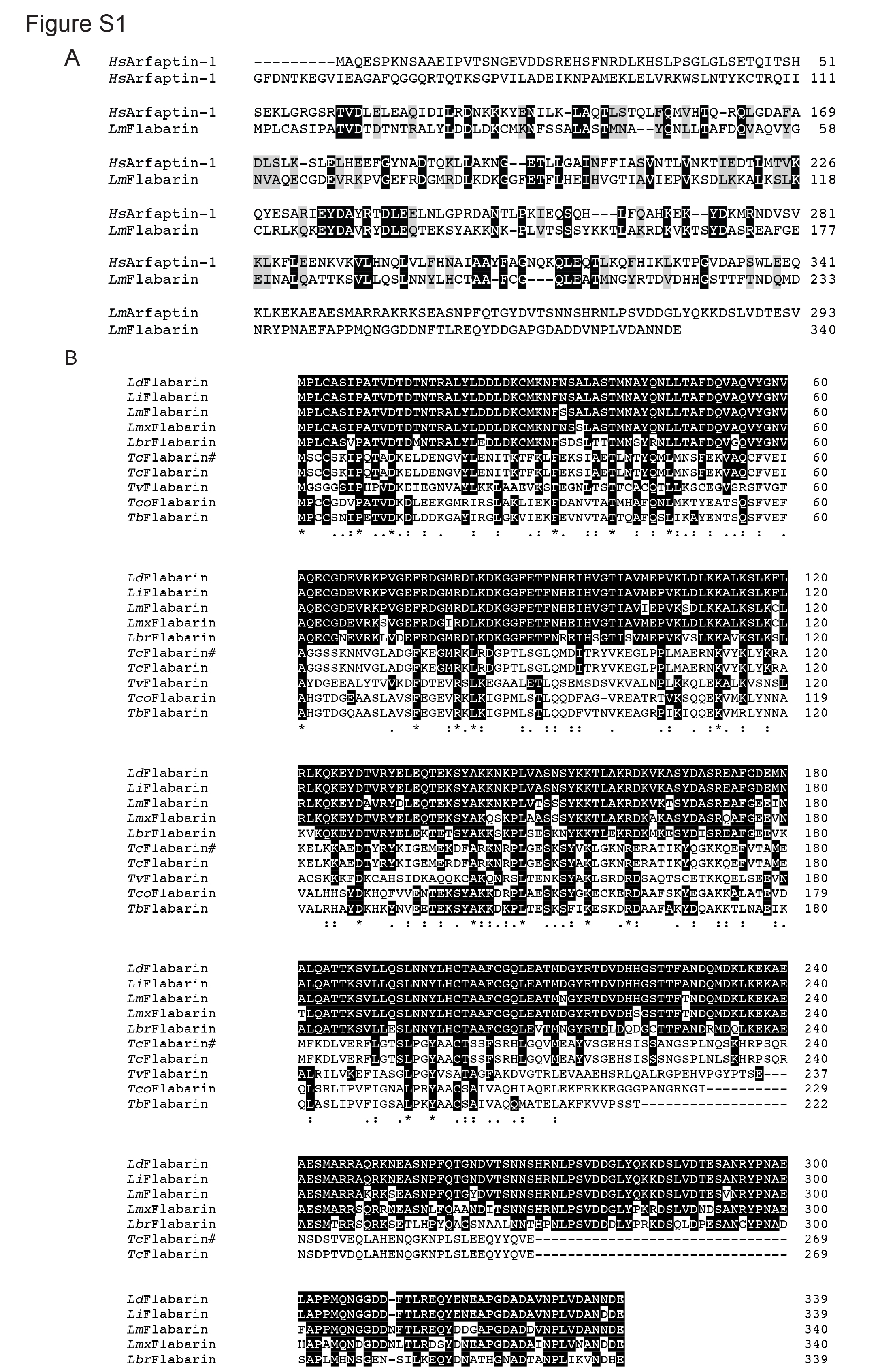

Supplement: Figure S1 — A: Sequence alignment between H. sapiens Arfaptin-1 and L. major Flabarin. Alignment of HsArfaptin-1 (Genbank U52521) and LmFlabarin (LmjF.27.1730) obtained from GeneDB [GeneDB-Blast-Lmajor, 2013 #13595]. Score = 84 (34.6 bits), Expect = 0.033, P = 0.032. Identities = 54/213 (25%), Positives = 88/213 (41%). Identical amino acids are highlighted in black, similar amino acids (apolar, polar) in grey. B: Sequence alignment of Ld Flabarin and its orthologues. Clustal W (1.83) (http://www.ch.embnet.org/software/ClustalW.html) alignment of LdFlabarin and its orthologues (Group OG5_148786, http://orthomcl.org): L. infantum (LinJ.27.1630), 99,7% id.; L. major (LmjF.27.1730), 90,3% id.; L. mexicana (LmxM.27.1730), 88,2% id.; L. braziliensis (LbrM.27.1860), 73,5% id.; Trypanosoma cruzi (TcCLB.506125.20 indicated by ♯, and TcCLB.504153.30), 20,3 and 19,8% id., respectively; T. vivax (TvY486_0013090), 19,2% id.; T. congolense (TcIL3000.11.2210.1), 18,3% id.; T. brucei (Tb927.11.2410, formerly Tb11.22.0001), 18% id. Two other orthologues, Tb427tmp.22.0001 (from another T. brucei strain) and Tbg972.11.2660 (T. gambiense), were not included because their amino acid sequences are identical to Tb927.11.2410. Identical amino acids are highlighted in black; *, and: correspond to “identity”, “semi-conservative substitution” and “conservative substitution”, respectively. (TIF) [file pone.0076380.s001.tif]

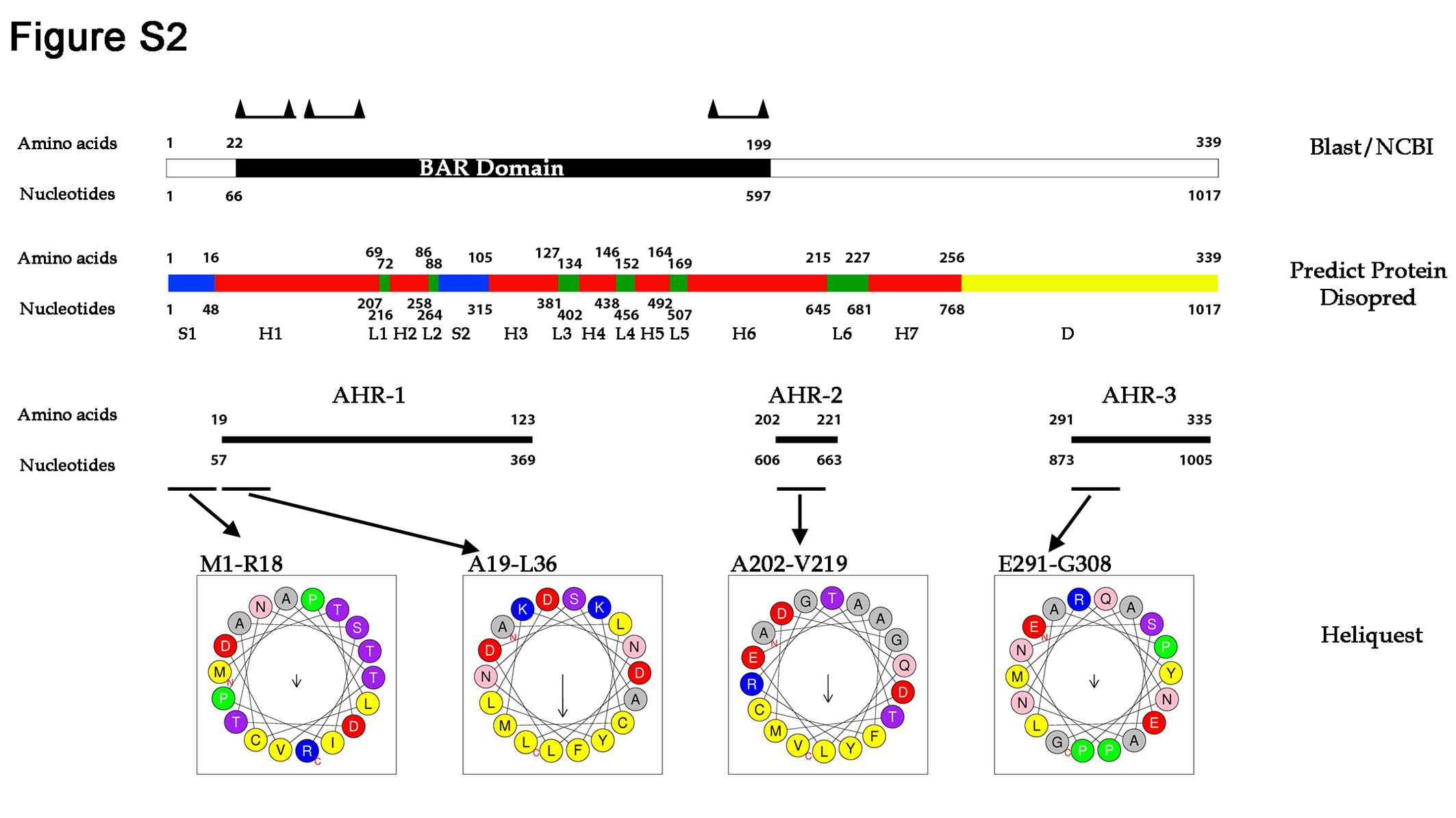

Supplement: Figure S2 — Analysis of structural domains and motifs of Ld Flabarin. Schematic representation of LdFlabarin structural domain predictions by NCBI-Blast (http://www.ncbi.nlm.nih.gov/blast/Blast.cgi), secondary structure predictions by PredictProtein (http://www.predictprotein.org/) and Disopred (http://bioinf.cs.ucl.ac.uk/disopred/), and potential amphiphilic helices by Heliquest (http://heliquest.ipmc.cnrs.fr). Amino acids are numbered starting with the first methionine. NB: (i) the α-helix H7 (227–256; PredictProtein) is considered as belonging to the disordered region (D) by Disopred and (ii) there may be a α-helix in the middle of the disordered region (PredictProtein). PredictProtein/Disopred: red is for α-helix (H), blue for β-strand (S), green for loop (L), and yellow for disordered (D); solid lines limited by solid triangles correspond to potential dimerization domains. Heliquest: AHR, amphiphilic α-helix region. The four selected 18-aa windows show α-helices as viewed from above: for a comparison, the first on the left (M1-R18) is not amphiphilic, the other three are; non-polar amino acids are yellow, grey, and green; polar amino acids are blue for cationic, red for anionic, and pink and purple for neutral. (TIF) [file pone.0076380.s002.tif]
