# Supplementary material for: LdFlabarin, a New BAR Domain Membrane Protein of Leishmania Flagellum
Source: PLoS One. 2013 Sep 27;8(9):e76380. doi: 10.1371/journal.pone.0076380 (PMC3785460; doi:10.1371/journal.pone.0076380)
Supplement: Table S1 — Potential modification sites of Flabarins and conservation between species. (DOC) [file pone.0076380.s003.doc]

**Table S1.** Potential modification sites of Flabarins and conservation between species

|  |  | *L. donovani/*  *infantum* | *L. major* | *L. mexicana* | *L. braziliensis* | *T. cruzi* | *T. vivax* | *T. congolense* | *T. brucei* |
| --- | --- | --- | --- | --- | --- | --- | --- | --- | --- |
| N-glycosylation^a^ | N267 | + | + | + | + | - |  |  |  |
| O-glycosylation^b^ | T225 | + | + | + | + | - | - | - |  |
| Palmitoylation^c^ | C4 | + | + | + | + | + | - | + | + |
| Protein tyrosine kinase^d^ | Y132 | + | + | + | + | - | - | - | - |
| AMPc/GMPc dependent  kinase^e^ | S286 | + | + | + | + |  |  |  |  |
| Casein kinase II^f^ | T48 | + | + | + | + | - | - | - | - |
|  | T217 | + | + | + | + | - | + | - | - |
|  | T260 | + | + | - | - |  |  |  |  |
|  | S275 | + | + | + | + | + |  |  |  |
|  | S286 | + | + | + | + |  |  |  |  |
|  | T313 | + | + | + | - |  |  |  |  |
| Protein kinase C^g^ | S116 | + | + | + | + | - | - | - | - |
|  | T129 | + | - | + | + | + | - | - | - |
|  | T137 | + | + | + | + | - | - | + | + |
|  | S153 | + | + | + | + | + | - | + | + |
|  | T185 | + | + | + | + | - | - | - | - |
|  | S269 | + | + | + | - | - |  |  |  |
|  | T313 | + | + | + | - |  |  |  |  |

Dark grey (+), conserved sites; light grey (-), non-conserved sites; white, absent (shorter protein sequence).

a: predicted by NetNGlyc (<http://www.cbs.dtu.dk/services/NetNGlyc/>) and Predict Protein (<http://www.predictprotein.org/>)

b: predicted by OGPET (<http://ogpet.utep.edu/main.php>)

c: predicted by CSS-PALM ([http://csspalm.biocuckoo.org](http://csspalm.biocuckoo.org/))

d, e, f, and g: predicted by Predict Protein (<http://www.predictprotein.org/>)

N, arginine; T, threonine; C, cysteine; Y, tyrosine; and S, serine.
